# Epidemiology and Regional Predictors of COVID-19 Clusters: A Bayesian Spatial Analysis Through a Nationwide Contact Tracing Data

**DOI:** 10.3389/fmed.2021.753428

**Published:** 2021-10-20

**Authors:** Kwan Hong, Sujin Yum, Jeehyun Kim, Daesung Yoo, Byung Chul Chun

**Affiliations:** ^1^Department of Preventive Medicine, Korea University College of Medicine, Seoul, South Korea; ^2^Graduate School of Public Health, Korea University, Seoul, South Korea; ^3^Transdisciplinary Major in Learning Health Systems, Department of Healthcare Sciences, Graduate School, Korea University, Seoul, South Korea; ^4^Veterinary Epidemiology Division, Animal and Plant Quarantine Agency, Gimcheon, South Korea

**Keywords:** COVID-19, spatial analysis, disease cluster, cluster analysis, risk factors, epidemiology

## Abstract

**Purpose:** Revealing the clustering risks of COVID-19 and prediction is essential for effective quarantine policies, since clusters can lead to rapid transmission and high mortality in a short period. This study aimed to present which regional and social characteristics make COVID-19 cluster with high risk.

**Methods:** By analyzing the data of all confirmed cases (14,423) in Korea between January 10 and August 3, 2020, provided by the Korea Disease Control and Prevention Agency, we manually linked each case and discovered clusters. After classifying the cases into clusters as nine types, we compared the duration and size of clusters by types to reveal high-risk cluster types. Also, we estimated odds for the risk factors for COVID-19 clustering by a spatial autoregressive model using the Bayesian approach.

**Results:** Regarding the classified clusters (*n* = 539), the mean size was 19.21, and the mean duration was 9.24 days. The number of clusters was high in medical facilities, workplaces, and nursing homes. However, multilevel marketing, religious facilities, and restaurants/business-related clusters tended to be larger and longer when an outbreak occurred. According to the spatial analysis in COVID-19 clusters of more than 20 cases, the global Moran's I statistics value was 0.14 (*p* < 0.01). After adjusting for population size, the risks of COVID-19 clusters were related to male gender (OR = 1.29) and low influenza vaccination rate (OR = 0.87). After the spatial modeling, the predicted probability of forming clusters was visualized and compared with the actual incidence and local Moran's I statistics 2 months after the study period.

**Conclusions:** COVID-19 makes different sizes of clusters in various contact settings; thus, precise epidemic control measures are needed. Also, when detecting and screening for COVID-19 clusters, regional risks such as vaccination rate should be considered for predicting risk to control the pandemic cost-effectively.

## Introduction

Revealing the transmission dynamics of coronavirus disease 2019 (COVID-19) is pertinent to ensure effective quarantine strategies, which are crucial to controlling the pandemic because of the limited medical resources worldwide (1). In particular, the clustering of highly contagious diseases such as COVID-19 is helpful to detect unknown characteristics of people or clusters that have a high transmission rate (2). Large clusters of COVID-19 lead to more rapid transmission and high mortality rates than sporadic cases since medical resources are limited to treat a certain proportion of the total population in a short period (1, 3). The characteristics of the infected population also matter when considering the transmission rate, and the fatality could differ between people (4). For instance, medical facilities or long-term health care service center-related clusters account for up to 36% of case fatalities (5, 6). In addition, familial or nosocomial clusters have also shown higher secondary attack rates than usual community settings (7). Therefore, apart from reviewing the epidemiological aspects of COVID-19 at an individual level, it is essential to analyze the epidemiology of COVID-19 clusters and identify the risk factors for the occurrence of clusters and make targeted quarantine strategies.

Combining contact histories of one case with other cases is essential to define and classify a COVID-19 cluster, a term that is heterogeneously used (8) but basically indicates two or more cases with known contact histories (3, 9, 10). Fortunately, South Korea investigates all contacts of COVID-19 cases based on various methods, including interviews, closed-circuit television (CCTV) footage, mobile global positioning system (GPS), credit card records, and quick response (QR) code-based entry logs for visitors and not only tests them but also actively quarantines close contacts for 14 days (11, 12). Therefore, we manually collated all cases in the first 6 months after the COVID-19 outbreak in South Korea and characterized their demographics through this data. Through constructing the infection tracks of transmission, clustering and classifying them were also possible.

A previous study on COVID-19 clusters provided valuable results, such as the exact transmission route or epidemiologic features (1, 13). However, the frequency or distribution of cases' demographics, which is essential in constructing effective methodologies for public health, in the other clusters may not be the same as that of the reported clusters. In addition, the risk factors for the formation of clusters may be other than the transmission of the virus itself, considering the differences in socioeconomic level or intervention intensity by region. For example, it is important to re-estimate the association of influenza vaccination and COVID-19 incidence, which showed negative associations in some ecologic studies (14), by adding herd immunity effect of regions to an individual's immunization status. Also, if the kinds of risk factors and their impact size on COVID-19 clusters are different from the individual level's one, it may lead to effective public health policymaking through controlling clusters in time. Furthermore, through spatial modeling, the prediction of COVID-19 clusters may be beneficial for the prevention of COVID-19 clusters in the near future as indicated in other studies (15–17). There was clear disparities of COVID-19 diagnostic testing and socioeconomic status or GDP by regions, including their geographical characteristics such as urbanized or connection levels. Among various analyzing methods, Bayesian methods are the most popular choice for spatial modeling since the spatial units are heterogeneous and have dependency at the same time, making it hard to evaluate relative effects of risk factors, which are also covariates to measure (18).

This study aimed to describe the characteristics and distribution of COVID-19 clusters at the national level. Furthermore, we intended to help establish effective quarantine strategies by identifying the risk factors for areas where COVID-19 clusters occurred. Lastly, we compared the predicted high-risk regions with the previous pandemic situation for convincing evidence.

## Materials and Methods

### Study Population

Information on 14,423 COVID-19 cases and all their investigated contacts was used in the study. The study period was from January 20, 2020, when the first confirmed case was identified in South Korea, to August 3, 2020. The government, and specifically the Korea Disease Control and Prevention Agency (KDCA), collected all data, for national COVID-19 pandemic control (11). The data of confirmed cases included age, gender, region on registration, symptom onset date, and classification as the cause of infection. The contact data included personal information of the cases and their identified contacts, contact dates, and places. The two data sets were linked to each other based on personal information. For comparison, public daily incidence count data and population data provided by KDCA (11) were collected by researchers until October 5, 2021.

**Figure 1:**
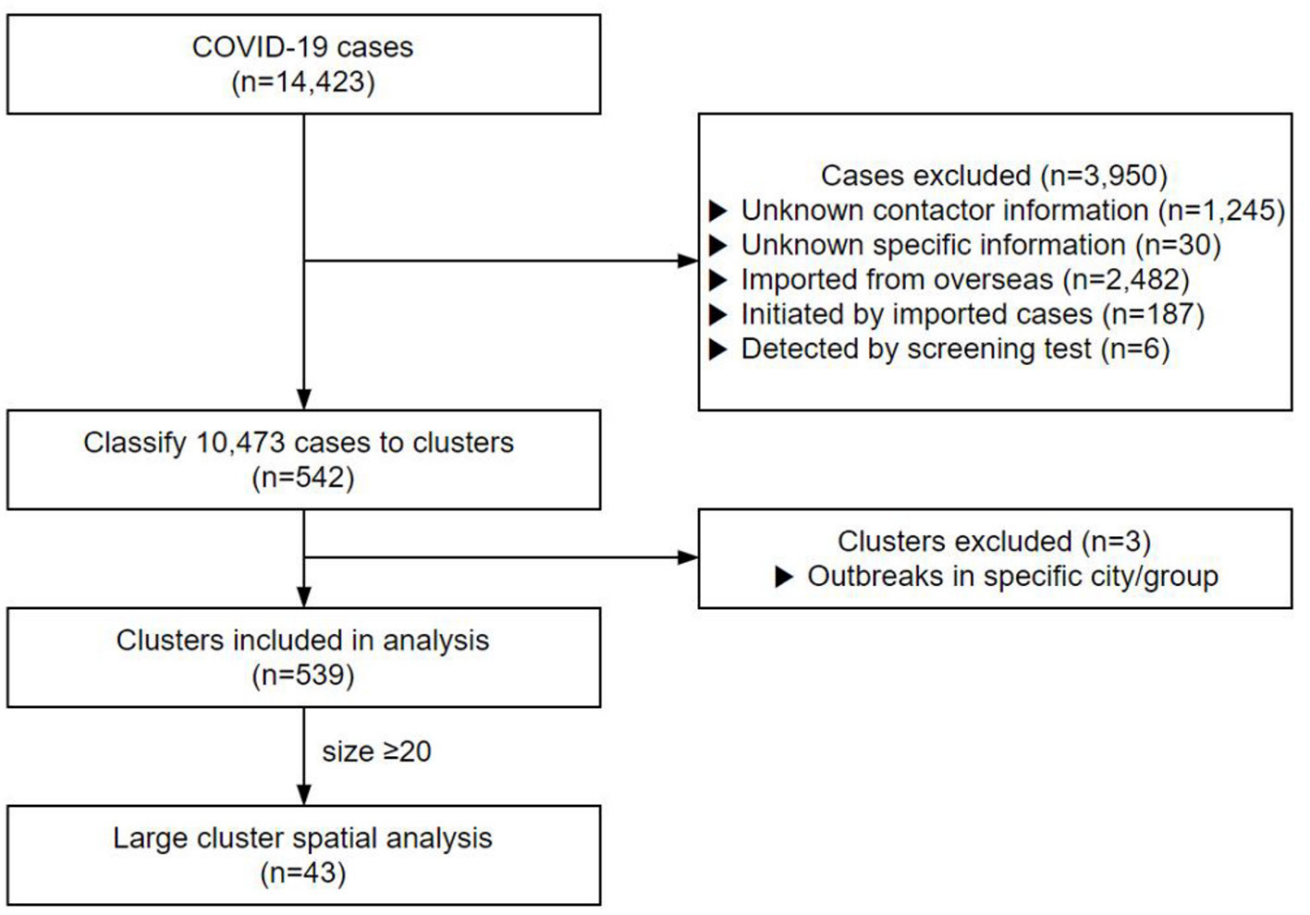
Study population included in the COVID-19 cluster analysis in South Korea.

Contacts with no clear personal information or repeated cases were excluded. A total of 1,245 cases with no contact data and 30 cases whose contact records were inaccurate were excluded from this study (Figure 1). In addition, 2,482 cases infected from abroad, 187 cases who contracted the infection from them, and six cases detected by screening tests were also excluded from the study. Finally, 10,473 cases were included in the clustering analysis.

### Ethics Statement

Since the data were collected as part of rapid response disease control by the government, informed consent was waived by the Korea University Institutional Review Board committee, and they granted an exemption for approval for this study (KUIRB-2020-0193-01). All study methods were carried out in accordance with the relevant guidelines and regulations.

### Definition of COVID-19 Cluster and Their Characteristics

A disease cluster in epidemiology is defined as a large medical event in a particular region and time period (4). Based on the exact contact histories, we defined the COVID-19 clusters as: (1) two or more cases with an exact contact history within 2 weeks or (2) cases from the investigated large clusters recorded by the KDCA. The size of the cluster was defined as the number of confirmed cases. The duration of the cluster was defined as the time interval between the earliest and the latest symptom onset dates. The selected region for each cluster was the highest frequency of cases among the included cases. All regions follow administrative boundaries, consisting of 250 districts in the Republic of Korea (19).

Contact places were used to classify the clusters, which were classified as detention centers or military units, education-related facilities, religious facilities like churches, restaurants or business-related facilities, medical facilities, multilevel marketing (house visiting sales), nursing homes, workplaces, and other community clusters. After classification, the mean size and duration of clusters based on the cluster characteristics were described and compared. In addition, the size, duration, and distribution of cluster characteristics were discussed using a timeline.

### Regional Risk Factors of COVID-19 Cluster

To reveal the regional risk factors for the formation of COVID-19 clusters, we divided regions according to whether they had (included) COVID-19 clusters with more than 20 people. Regional factors were derived from the Korean Statistical Information Service (KOSIS) and Community Health Survey (CHS) provided by the KDCA, including the following factors: financial independence index (higher is more independent, 0–100); a number of doctors per 1,000 population; health screening test receiving rate (%); gender ratio (women: men); population; influenza vaccination rate in the previous year; the diagnosed proportion of hypertension or diabetes mellitus; physical activities (moderate, more than three times per week); active smoker proportion; alcohol consumption (more than three times per week); the proportion of the married population; hand washing habits after outdoor activities; number of family members; household income (>50,000,000 won); the proportion of the employed population; level of education (higher than college); basic livelihood security recipients; and the experience of unmet medical needs (20).

### Statistical Analysis

To determine an appropriate spatial model, spatial autocorrelation of COVID-19 clusters was tested using the global Moran's I statistics with 999 Monte Carlo simulations (21). The *k*-nearest neighbor (number of neighbors: 3)-based method was used as the distance criterion for Moran's *I*-test and further weight matrix was used in conditional autoregressive models (22). We selected possible risk factors by univariate logistic regression analysis with a higher risk of having a COVID-19 cluster (*p*-value < 0.2). The non-spatial multivariate model with the stepwise-selected variables (*p*-value < 0.1) using logistic regression analysis was defined. After estimating spatial autocorrelation of residuals for the non-spatial multivariate model, we constructed the final spatial model. Spatial and non-spatial random effects were added by the Besag, York, and Mollié (BYM) model, in which regions with clusters of more than 20 satisfied conditional distributions (23, 24). We used Bayesian inference for the parameter estimation, and flat priors were used as a prior distribution for covariates, and Gamma distributions with an extensive range (0.01) were used as a prior distribution for a variance for the spatial or non-spatial residual terms (25). The deviance information criterion (DIC) (26) was compared for the final model selection. Estimated mean values of parameters were used for the visualization of cluster mapping. After mapping the predicted probabilities of COVID-19 clusters, we compared the result with the actual regions with more than 20 COVID-19 cases, incidence per 1,000,000 population, and local Moran's I statistics (27) 2 months after the study period, from August 5, 2020, to October 5, 2020.

Shape files for a base map of South Korea by administrative regions were open-source data and were downloaded through the Korea National Spatial Data Infrastructure Portal, which is available for free (28). Packages named spdep, R2WINBUGS, ggplot2, and CARBayes in R software (version 4.0.3; R Foundation for Statistical Computing, Vienna, Austria) were used for data management, analysis, and visualization.

## Results

### Study Population

Of the 14,423 cases up to August 3, 2020, 10,473 cases were classified into 542 clusters based on their contact data (Figure 1). We excluded the cluster that was classified as a particular religion (e.g., Shincheonji) (29) and that was classified as visiting a particular region with a high incidence of COVID-19 (e.g., Daegu, Gyeongbuk) (30) since they were screened without exact contact histories. After exclusion, we analyzed a total of 539 clusters. The cluster's mean size was 19.21, and the mean duration was 9.24 days. Of the total 4,936 cases, 2,253 were males (45.6%) and 2,683 females (54.4%), with a mean age of 52.2 years.

### Characteristics of COVID-19 Clusters

Clusters were classified into nine types, as shown in Table 1. Except for community clusters (*n* = 407), the number of clusters was high (in decreasing order) in medical facilities (*n* = 37), workplaces (*n* = 28), nursing homes (*n* = 20), and religious facilities (*n* = 17), but the mean cluster size was large in multilevel marketing (86.1 cases), restaurants/business-related (41.2 cases), and religion-related (31 cases) clusters. The mean duration for cluster formation was ≥3 weeks in multilevel marketing and medical facilities, while religious facilities and workplaces took ≥2 weeks (Supplementary Figure 1). Multilevel marketing, religious facilities, and restaurants/business-related clusters tended to be larger and longer when an outbreak occurred.

**Table 1:** Overall description of COVID-19 clusters.

**Cluster category**	**Total (*n*)**	**Duration (day)**	**Mean size (person)**	**Cluster size (** ***n*** **)**									
				**2–5**	**6–9**	**10–19**	**20–29**	**30–39**	**40–49**	**50–99**	**100–149**	**150–199**	**200–299**
Buildings/offices	6	17.5	19.7	–	3	–	1	2	–	–	–	–	–
Communities	407	6.2	3.5	375	20	8	–	2	–	1	1	–	–
Detention centers/military units	5	9.0	8.6	2	2	–	1	–	–	–	–	–	–
Educational facilities	5	8.6	5.2	3	1	1	–	–	–	–	–	–	–
Medical facilities	37	21.6	28.2	15	5	7	–	2	1	3	3	1	–
Multilevel marketing	6	27.3	86.2	–	1	1	–	–	1	1	–	1	1
Religious facilities	17	17.9	31.3	1	3	3	3	1	4	1	1	–	–
Restaurants/business establishments	8	9.4	41.3	2	3	2	–	–	–	–	–	–	1
Nursing homes	20	18.1	16.8	7	2	4	4	1	1	1	–	–	–
Workplaces	28	15.4	20.4	8	8	9	–	1	–	–	–	2	–
**Total**	**539**	**9.1**	**9.2**	**413**	**48**	**35**	**9**	**9**	**7**	**7**	**5**	**4**	**2**

*Bold values refer to “total”*.

Educational facilities had a higher percentage of small clusters of five or less, and multilevel marketing had the highest percentage of more than 100 clusters, especially those with more than 200 cases (Supplementary Figure 2). Medical facilities, restaurants/business establishments, and workplaces had a high percentage of small clusters, but the percentage of large clusters was also high, with a large deviation. Clusters in multilevel marketing and religious facilities were distributed in various sizes. The regional distribution of the mean duration and size of the clusters is shown in Supplementary Figure 3. Overall, the mean duration of clusters was long in the Seoul metropolitan and Kyongsang-do areas, and their mean cluster size was also significant.

### Regional Risk Factors of COVID-19 Cluster

As a result of the global Moran's I test, regions with COVID-19 clusters with more than 20 cases had positive spatial autocorrelation (*p*-value < 0.01, Moran's I statistics of 0.14), implying that the nearby regions had a similar status of occurrence of a COVID-19 cluster.

The identified regional risk factors by Bayesian inference with 30,000 iterations and 10,000 burn-ins using the Besag, York, and Mollié (BYM) spatial model is shown in Table 2. When analyzing the regional risk factors of COVID-19 clusters with more than 20 cases by region, a lower mean age [odds ratio (OR) = 0.95, 95% credible interval (CI): 0.87–1.04], male gender (OR = 1.29, 95% CI: 1.09–1.56), low influenza vaccination rate (OR = 0.87, 95% CI: 0.77–0.96), low health screening receiving rate (OR = 0.95, 95% CI: 0.87–1.04), and slightly low household income (OR = 1.00, 95% CI: 0.99–1.00) were associated with a higher risk of having COVID-19 clusters. Convergence is evaluated visually and statistically. Trace plots of each variable are shown in Supplementary Figure 4. The results of the Gelman-Rubin convergence diagnostics were 1.06 overall, without exceeding 1.1 in any variable.

**Table 2:** Odds ratios and 95% credible intervals of having COVID-19 clusters with more than 20 cases by Bayesian conditional autoregressive (CAR) model.

**Factors**	**Odds ratio**	**2.5%**	**97.5%**
Mean age	0.95	0.87	1.04
Gender (male)	1.29	1.09	1.56
Influenza vaccination rate in 2019 (%)	0.87	0.77	0.96
Health screening test rate (%)	0.95	0.87	1.04
Income (more the 5 million won)	1.00	0.99	1.00

*Deviance information criterion: 169.9*.

### Predicted COVID-19 Clusters and the Cumulative Incidence

**Figure 2:**
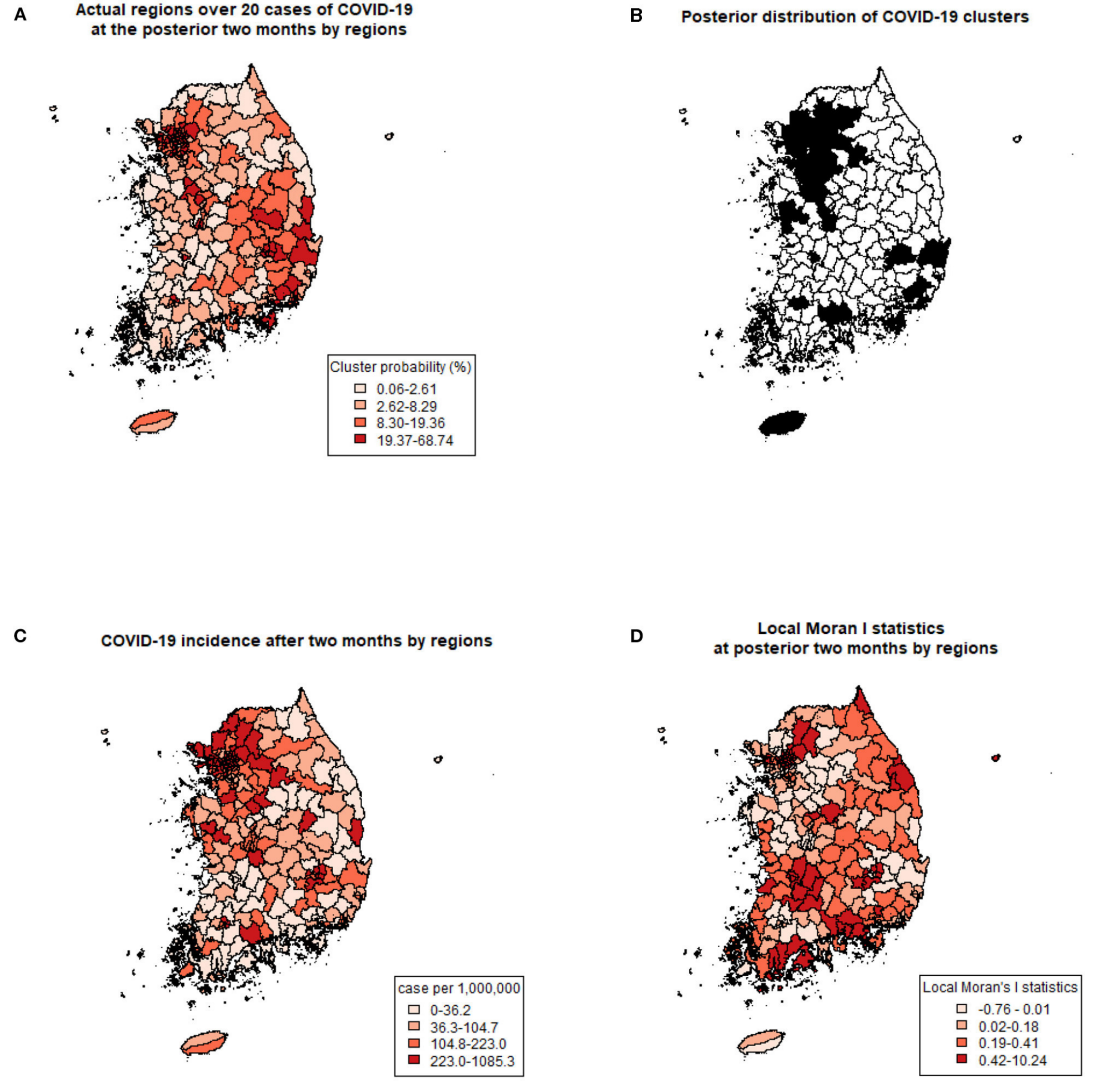
Comparing predicted clusters and actual COVID-19 with three criteria. **(A)** Prediction of COVID-19 clusters by posterior probabilities of the spatial cluster model. **(B)** Actual regions with more than 20 COVID-19 cases at the posterior 2 months of the study period. **(C)** COVID-19 incidence (number of cases per 1,000,000) after 2 months of the study period. **(D)** Local Moran's I statistics at posterior 2 months of the study period.

Using previously revealed risk factors, the predicted probability of COVID-19 clustering by regions were displayed (Figure 2A) and compared with the actual regions with more than 20 COVID-19 cases after 2 months of the study period (Figure 2B). Predicted probabilities of COVID-19 clusters showed similar patterns with actual regions with over 20 cases. Also, the incidence (a number of COVID-19 cases per 1,000,000 population) during the same period is shown in Figure 2C. Since the cumulative incidence of COVID-19 in this period was released publicly by only 228 administrative regions, which were different from the collected data used in this study, direct comparison by each region was not possible. Instead of a direct comparison, we calculated local Moran's I statistics for the later period (Figure 2D). A positive I indicates that neighboring features are similar, regardless of the size of the response value. Regions with higher similarities were well-matched with posterior distribution of COVID-19 cluster probabilities.

## Discussion

This study found that the size and duration of the COVID-19 cluster depended on the cluster's characteristics by contact history-based clustering. Specifically, clusters related to medical facilities, workplaces, nursing homes, and religious facilities were frequent. Also, clusters related to multilevel marketing, restaurants/business, and religious facilities were more prominent than others. In addition, the transmission in facilities related to multilevel marketing, medical facilities, religious facilities, and workplaces continued for more than 2 weeks. Clearly, places with frequent contact with non-specific people in long time intervals increase cluster formation. Therefore, preventive measures for COVID-19 should include intensive management in facilities related to medicine and religion, where cluster formation was easy and long-spreading, and in multilevel marketing and restaurants/business-related facilities, which did not have many clusters but could result in large COVID-19 clusters. Moreover, multilevel marketing, which had the highest cluster occurrence rate of more than 200 cases, is reported to have similar characteristics to that of religious clusters (31); a careful approach is needed to achieve quarantine results for such high solidarity groups.

By screening these high-risk clusters and applying quarantine policies, efficient quarantine can be expected; however, stigma may arise for certain clusters (32). Recently, there has been a nationwide mass infection in Korea from certain religious-related facilities, and the representative of that religious association has issued an apology (30). It is necessary to be careful not to develop targeted quarantine for efficiency in public health and economic aspects into an aversion to specific targets. It was difficult to compare the community clusters to other clusters directly. Community clusters might encompass large clusters that may exist but have not been identified yet. Even so, common causes of infection resulted in such clusters, and 28 clusters (6.9%) in this classification had over five cases. Therefore, community clusters may also be controlled through effective quarantine measures. Through targeted policies based on scientific evidence through spatial analysis, we could increase compliance and effectiveness of local governments and citizens in the daily practices of public health. Regarding establishing an effective quarantine policy, it may be more logical and appropriate to take a regional approach than to focus on individual risk factors.

This is the first study to classify all COVID-19 cases into clusters to identify their characteristics and the risk factors of the regions with COVID-19 clusters. Since the COVID-19 cluster showed spatial autocorrelation, it is necessary to consider spatial models rather than conventional regression models for the risk factor analysis. Furthermore, applying Bayesian inference in spatial modeling was crucial since the neighboring regions showed dependency and heterogeneity simultaneously, making it hard to estimate the later distribution of COVID-19 in a small-area with a frequentist approach (18).

The risk of clusters was higher in regions with more males, a low mean age of the population, low influenza vaccination rate over the past year, low health screening test receiving rate, and low household income. In particular, the odds ratio of influenza vaccination, which is still controversial about its protective effect on COVID-19 infection (14, 33), showed narrow credible intervals, indicating a possible association between the two factors. One possible explanation is that the vaccinated population gains T-cell diversities (34), leading to a protective effect on COVID-19 infection. Gender differences in COVID-19 clustering were not actively reported, but one study (35) showed that males was more vulnerable to death and ICU admission because of COVID-19. Likewise, the male gender seems to have more risk to occurrence of COVID-19 cluster in our study, possibly because males are more likely to have outdoor occupations or social meetings than females. Other revealed risks include lower mean age, lower health screening test receiving rate, and lower mean household income showed credible intervals, indicating that further studies are needed. Older age was a major risk factor in previous research (14), especially at an individual level (6). However, considering that social contacts usually occur frequently in young populations (13), the direction of risk of age seems appropriate. In South Korea, since health screening is recommended with the national insurance program, health screening tests are periodically performed in adults (12). Therefore, low health screening test receiving rate may indicate that an individual is finding it hard to receive health resources, which is similar to low socioeconomic status, which was discussed in studies from New York City (15) and Nigeria (16). The relationship between socioeconomic status and COVID-19 incidence is still in debate in the spatial aspect; therefore, we need further measurements and estimations of the effect size and direction of socioeconomic status in regional COVID-19 incidence. Since this study is the only study that showed odds ratio between regions, further spatial studies are needed to confirm the association at the community level. General risk factors of COVID-19 at the individual level are discussed actively (35–37), but the risk factors resulting in COVID-19 clusters in specific regions have rarely been studied. The regional factor should be considered in the analysis of COVID-19 clusters. Moreover, spatial modeling, including the spatial autoregressive effect, should be applied to exclude the regional effect of COVID-19 when analyzing spatial data.

This study also has some limitations. First, due to the limited data available, we were unable to match some cases that were confirmed later in the analysis period to their contacts. However, the data of ~14,000 cases by early August 2020 were utilized, which were sufficient for analysis. We also compared the predicted clusters through regional risks visually with the actual incidence and patterns of COVID-19 at the posterior 2 months. Second, due to insufficient contact tracing data, it was impossible to analyze if there were not more than two cases with the same causation. In South Korea, epidemiological investigations are conducted from 2 days before the onset of symptoms until quarantine, including not only close contacts but also all daily contacts. Therefore, it was impossible to identify the contacts whose transmission duration was longer than the range of investigation or who had not been identified through CCTV footage and credit card records. Third, in our study, we evaluated regions with the highest number of cases in the cluster; however, clusters spread over multiple regions may have differences in the risk factors with these clusters.

By cluster analysis and spatial modeling, we discovered the characteristics of COVID-19 clusters and the risks of COVID-19 clusters. COVID-19 clusters related to medical facilities, workplaces, nursing homes, and religious facilities were frequent, and those related to multilevel marketing, restaurants/business, and religious facilities were larger than others. Clusters over 20 cases were spatially correlated, and the risk factors for the occurrent were lower mean age, male gender, low influenza vaccination coverage, low health screening test receiving rate, and low mean household income.

Likewise, clustering COVID-19 cases should be retrospectively performed and analyzed for effective COVID-19 quarantines. We believe that our results could help control regional risks to predict COVID-19 vigilance and other similar respiratory viruses in the future. The direction and methodology for this regional risk factor analysis may be extended to other nations for effective cluster control and future epidemics by applying the spatial approach to deal with an ongoing communicable disease.

## Data Availability Statement

The original contributions presented in the study are included in the article/Supplementary Material, further inquiries can be directed to the corresponding author.

## Ethics Statement

The studies involving human participants were reviewed and approved by Korea University Institutional Review Board Committee. Written informed consent for participation was not required for this study in accordance with the national legislation and the institutional requirements.

## Author Contributions

KH, SY, and BC performed material preparation, data collection, and analysis. The first draft of the manuscript was written by KH and SY prepared Figures 1, 2. All authors contributed to the conception, design of the study, commented on the previous versions of the manuscript, read, and approved the final manuscript.

## Funding

This work was supported by the Research Program funded by the Korea Centers for Disease Control and Prevention (Grant Number 2020-ER5313-00). The Korea Centers for Disease Control and Prevention provided the data and funding but did not analyze data or write a manuscript.

## Conflict of Interest

The authors declare that the research was conducted in the absence of any commercial or financial relationships that could be construed as a potential conflict of interest.

## Publisher's Note

All claims expressed in this article are solely those of the authors and do not necessarily represent those of their affiliated organizations, or those of the publisher, the editors and the reviewers. Any product that may be evaluated in this article, or claim that may be made by its manufacturer, is not guaranteed or endorsed by the publisher.
