# Supplementary material for: Epidemiology and Regional Predictors of COVID-19 Clusters: A Bayesian Spatial Analysis Through a Nationwide Contact Tracing Data
Source: Front Med (Lausanne). 2021 Oct 20;8:753428. doi: 10.3389/fmed.2021.753428 (PMC8563697; doi:10.3389/fmed.2021.753428)
Supplement: Supplementary file 1 [file Data_Sheet_1.docx]

Supplementary Material

# Supplementary Figures and Tables

## Supplementary Figures


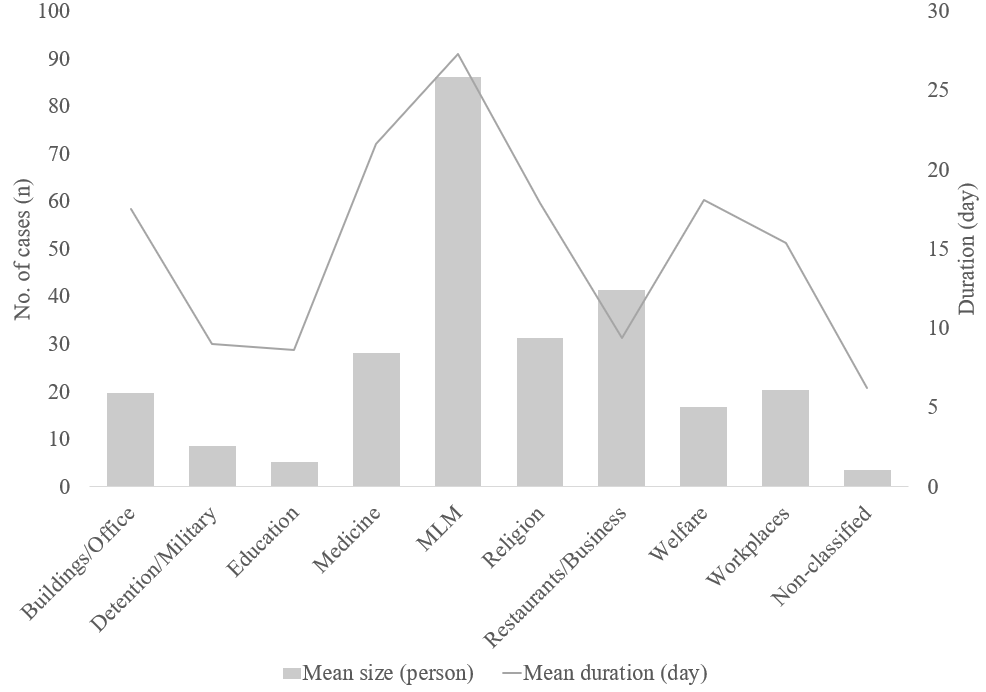


**Supplementary Figure S1.** Mean size and duration of COVID-19 clusters. MLM, Multilevel marketing


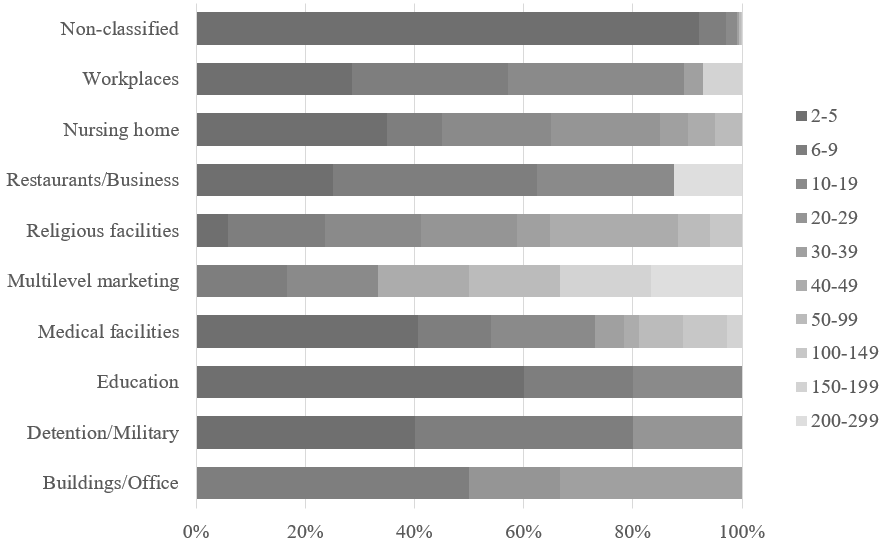


**Supplementary Figure S2.** Distribution of cluster size by COVID-19 cluster classifications

**Supplementary Figure S3.** Mean COVID-19 cluster duration and size by regions


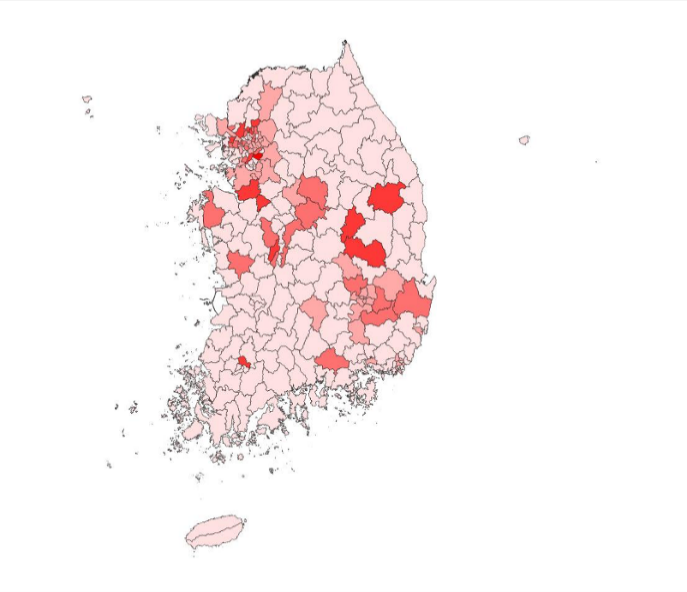

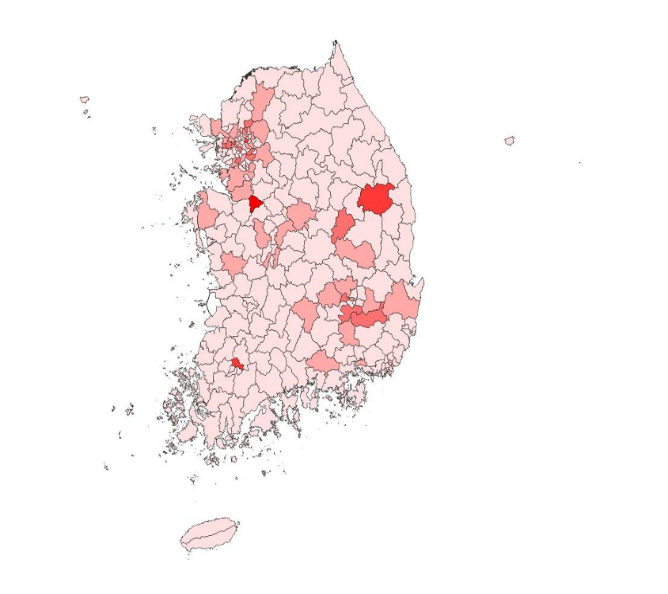


(A)

(B)

1. Mean COVID-19 cluster duration by regions
2. Mean COVID-19 cluster size by regions


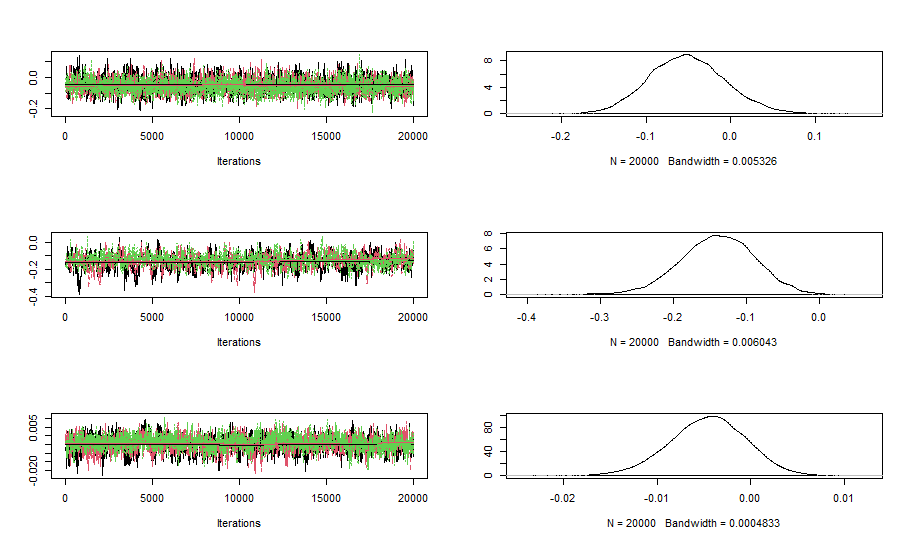

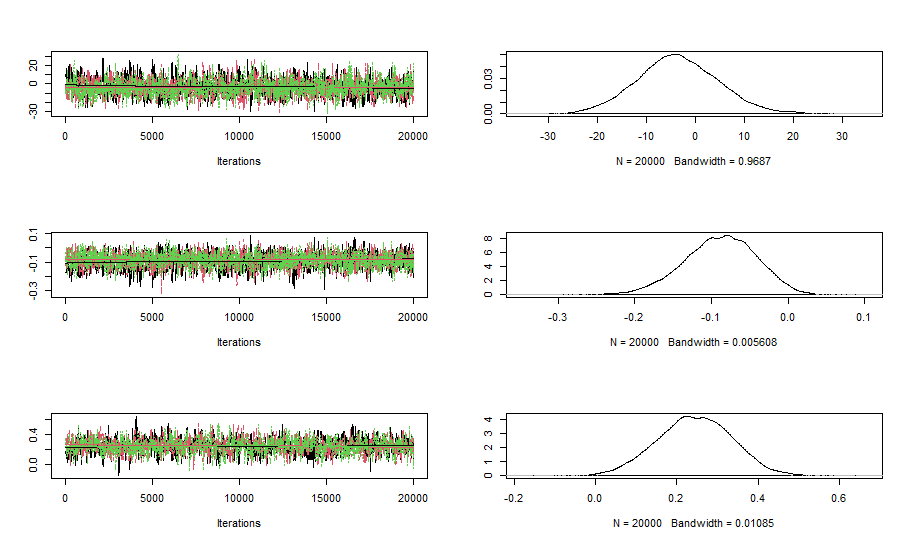


**Supplementary Figure S4.** Trace plots for the Bayesian spatial model used in this study

Intercept, sex ratio, mean age, health screening test receiving rate, influenza vaccination rate, and household income level are shown in serial order.
